# Predictors and reasons for epilepsy patients to decline surgery: a prospective study

**DOI:** 10.1007/s00415-022-11510-3

**Published:** 2022-12-06

**Authors:** Mirja Steinbrenner, Tabea Tito, Christoph Dehnicke, Martin Holtkamp

**Affiliations:** 1 Department of Neurology and Experimental Neurology, Epilepsy-Center Berlin-Brandenburg, Charité–Universitätsmedizin Berlin, Campus Benjamin Franklin, Hindenburgdamm 30, 12200 Berlin, Germany; 2 Epilepsy-Center Berlin-Brandenburg, Institute for Diagnostics of Epilepsy, Ev. Krankenhaus Königin Elisabeth Herzberge, Berlin, Germany

**Keywords:** Drug-resistant epilepsy, Epilepsy surgery, Presurgical assessment, Quality of life

## Abstract

**Background:**

In patients with drug-resistant focal epilepsy, resective surgery is the most successful treatment option to achieve seizure freedom. However, a surprisingly high rate of patients declines their physicians’ recommendation to undergo removal of the seizure focus or—if necessary—further video-EEG monitoring (VEM).

**Methods:**

In this prospective study, consecutive patients in presurgical assessment with at least one scalp VEM between 2016 and 2018 were included. We assessed both epilepsy-related and psychosocial variables as well as decision-making of physicians and patients, including reasons for decline in the latter.

**Results:**

Out of 116 patients with a total of 165 VEM, 20 patients were eventually found to be ineligible for resection, 51 declined, and 45 agreed on recommendations for resection or further VEM diagnostics. Patients most frequently declined due to general fear of brain surgery (*n = *30, 59%) and currently lower seizure frequency (*n = *11, 22%). An independent predictor of patients’ decline was less epilepsy-related fear (OR 0.43; *p = *0.02) assessed in a standardised questionnaire.

**Conclusion:**

Half of the patients potentially eligible for resective surgery decline the operation or further VEM procedures. Patients who decline are more fearful of brain surgery than of ongoing disabling seizures. More insight is needed to improve counselling of patients.

**Supplementary Information:**

The online version contains supplementary material available at 10.1007/s00415-022-11510-3.

## Introduction

In one-third of patients, focal epilepsy is drug-resistant, i.e., seizures persist despite at least two trials with adequate antiseizure medication. For this group, resective surgery is the best option to achieve seizure freedom, rendering 64% of patients seizure-free [1] and improving postoperative quality of life [2, 3]. Surprisingly, retrospective analyses have shown that 20–32% of patients in presurgical assessment decline the recommendation to undergo resective surgery [4, 5] and up to 50% decline intracranial EEG recordings after scalp EEG was not meaningful [6]. Predictors of patients’ rejection of surgery comprise learning disability, normal (“negative”) MRI, bilateral lesions on MRI, extratemporal epilepsy, psychiatric comorbidities, and being employed [4, 7, 8]. Intellectual disability was independently associated with patients’ or care givers’ refusal of intracranial EEG [6]. One small prospective study (*n = *32) found that epilepsy patients who declined surgery had a lower perceived disease burden, expressed more fear about surgery, rated the benefit of information given by others lower and had higher incidences of psychiatric comorbidities [9].

In this prospective study, we aimed to gain further insight into characteristics and individual reasons of patients with epilepsy declining their physicians’ recommendations for resection or further video-EEG monitoring (VEM) during presurgical assessment.

## Methods

### Study population

All consecutive patients with drug-resistant focal epilepsy who underwent presurgical assessment at the Epilepsy-Center Berlin-Brandenburg, Germany, between 1st January 2016 and 31st December 2018 were screened for inclusion.

Inclusion criteria were at least one scalp VEM during the study period, age ≥ 18 years, adequate German language skills, and mental capabilities to understand and fill out self-assessment questionnaires. Exclusion criteria were diagnosis of psychogenic non-epileptic seizures (PNES) only and premature discontinuation of presurgical assessment.

### Presurgical assessment

Presurgical assessment at the Epilepsy-Center Berlin-Brandenburg has been described in detail in previous work [6]. In short: After standard diagnostic procedures including scalp VEM, high-resolution MRI (3T) following an epilepsy-specific protocol, neuropsychological testing, and findings of each patient are discussed in an interdisciplinary epilepsy surgery meeting. The team then determines non-feasibility of surgery, e.g., due to multifocal epilepsy or overlap with eloquent areas, or recommends either direct resective surgery, further scalp, or intracranial VEM diagnostics. The latter includes implantation of hemispheric epidural peg, foramen ovale, subdural or depth electrodes either alone or in combination according to clinical necessity. During the study period, recommendations were explained to all patients by the same senior neurologist (C.D.). These face-to-face conversations were either held directly at the end of patients’ in-hospital stay or, in case of outstanding diagnostic results, in a separate meeting on-site (usually 2–3 weeks after hospital discharge). Based on individual results of diagnostic procedures and additional clinical characteristics, patients were counselled on their expected individual chance on postoperative seizure freedom, individual peri-operative and long-term postoperative risks. Furthermore, patients were informed on their individual SUDEP risk, in particular if they suffered from focal to bilateral tonic–clonic seizures. For further information to the patients, we used a self-designed information sheet written in easy-to-understand language explaining in detail the procedures of epilepsy surgery and intracranial EEG recordings. All patients were offered an additional counsel meeting with the neurosurgeon.

The patients that declined their neurologists’ recommendation were asked in an open format to explain their decision. We later grouped the responses into the following categories: (1) general fear of brain surgery, (2) fear of peri- or postoperative complications, (3) assumed low probability of postoperative seizure freedom, or (4) currently perceived low seizure frequency. With respect to decisions for or against resections or further VEM, a follow-up period until 31st of December 2019 was considered.

Additionally, standard clinical variables including psychiatric comorbidities, results of all diagnostics done during presurgical assessment, as well as seizure outcome 1 year after surgery and after last VEM in those not resected using the ILAE (International League against epilepsy) outcome scale [10] were assessed.

All included patients were asked to fill out the PESOS questionnaire (PESOS = Performance, Socio-demographic aspects, Subjective evaluation), a self-assessment for patients with epilepsy asking for disease-specific constraints. In the current study, we included the categories “restrictions in daily life” (14 subitems) and “epilepsy related-fear” (11 subitems) as well as the item “overall satisfaction with current therapy” [11].

### Statistics

Statistical analysis was done using IBM SPSS Statistics 26. Binary regression analysis (inclusion method: stepwise backward; *p < *0.1 [*p* in], *p < *0.05 [*p* out]; iteration 20; cut-off set at 0.5; constant included) was performed to calculate odds ratios (ORs) with 95% confidence intervals as estimates for variables independently predicting whether patients did not adhere to the given recommendation. Statistical significance was set at *p < *0.05.

## Results

### Study population

During the study period, 211 patients were screened for inclusion. Overall, 47 patients had to be excluded: 15 were younger than 18 years, 22 had an IQ < 70, seven patients were not drug-resistant, and three curtailed their presurgical assessment prematurely, leaving 164 eligible patients for further analysis. Forty-eight patients declined to participate in the study, and thus, 116 patients were included.

Almost half of the included patients were female (*n = *55; 47%); at last scalp VEM, median age was 32 years (IQR 27–41) and median duration of epilepsy 15 years (IQR 8–23). Including all available clinical, EEG and neuroimaging information, 58 patients (50%) had clear temporal and 22 (19%) had extratemporal lobe epilepsy; in another 36 (31%), seizure origin remained unknown. For further clinical variables, see Supplemental Material Table 1.

**Table 1:** Patients’ decision against recommendations for resection or further VEM diagnostics

	Patients’ decision^a^		OR	95% CI	*p* value
	Decline	Agreement			
*n*	51	45			
Sex, *n* (%)					
Female	25 (49)	22 (49)	1.000		
Male	26 (51)	23 (51)	2.178	0.781–6.071	0.137
Age at last scalp VEM, years (median, IQR)	33 (29–44)	32 (24–38)	0.957	0.915–1.000	0.051
Presence of psychiatric comorbidity, *n* (%)					
Not present	37 (73)	29 (64)	1.000		
Present	14 (27)	16 (36)	0.405	0.141–1.165	0.094
Employment status, *n* (%)^b^					
Unemployed	24 (47)	23 (51)	1.000		
Employed	27 (53)	22 (49)	0.752	0.268–2.109	0.588
Restrictions in daily life, score (median, IQR)	23 (10–46)	39 (28–53)	0.834	0.428–1.622	0.592
Epilepsy related-fear, score (median, IQR)	30 (12–42)	36 (24–59)	**0.428**	**0.210—0.873**	**0.020**
Overall satisfaction with current therapy, *n* (%)					
Satisfied	24 (47)	19 (42)	1.000		
Unsatisfied	17 (33)	11 (24)	1.886	0.520—6.842	0.335

For independent predictors of patients’ decline of the given recommendation, the last decision made during the study period by each patient that either led to resection, further diagnostic VEM, or end of presurgical assessment was included.

*OR* odds ratio; *95% CI* 95% confidence interval; *n* number; *IQR* interquartile range

Bold values indicate the statistical significant (*p* < 0.05)

^a^The regression model was calculated for *n = *82 patients, as PESOS was missing or incomplete in 14 patients

^b^To dichotomize the variable employment status, we subsumed patients’ profession (see Supplementary Material Table 1 for more details): employed includes employees, students, trainees and public servants; unemployed includes job-seekers and people without own income (dependent on partner’s/ families’ income working in sheltered workshop, reduced earning capacity pension)

Eventually, 35 patients (30%) underwent resective surgery, 13 of those directly after scalp VEM and 22 after intracranial VEM. One year after surgery, two-thirds of patients (66%) had a good postoperative outcome without disabling seizures (ILAE 1 + 2) (Supplemental Material Table 1). Of the patients who did not undergo resection (*n = *81), nine (11%) were free of disabling seizures 1 year after their last VEM (*p < *0.001).

In the category “restrictions in daily life” of the PESOS questionnaire, nine patients (21%) of those that had declined (*n = *43) and ten patients (25%) of those that had agreed (*n = *40) to the given recommendation scored into the categories “very strong” or “strong”; for “epilepsy-related fear”, two patients (5%) of those that had declined (*n = *43) and five (12%) that had agreed (*n = *40) scored in the category “strong”. For further details, see Supplemental Material Table 2.

### Decision-making

**Fig. 1:**
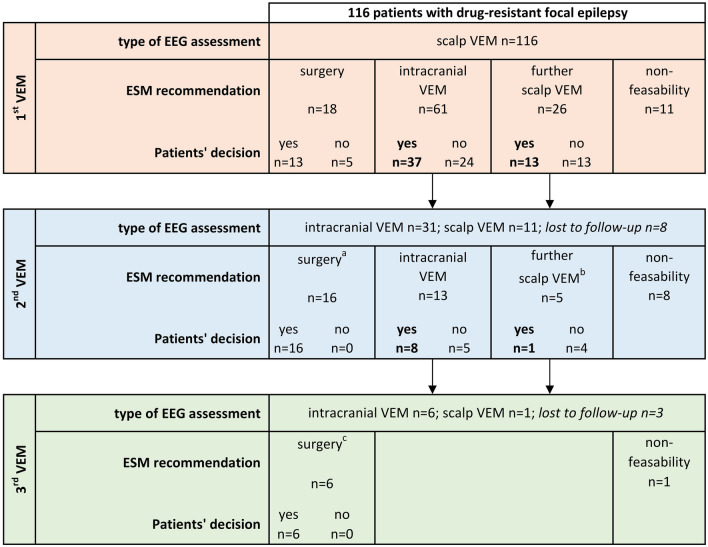
Overview of patient’s clinical pathways and decision-making during presurgical assessments. *EEG* electroencephalogram; *ESM* interdisciplinary epilepsy surgery meeting; *VEM* video-EEG monitoring; intracranial EEG comprises recordings with foramen ovale, epidural peg, subdural and/or stereotactic electrodes; non-feasibility: surgery not possible due to multifocality/overlap with eloquent areas/non-localisable focus. Patients were lost to follow up when they had previously agreed on a recommendation but eventually did not undergo the planned procedure during the study period. ^a^13 had subdural or depth electrodes, 3 had foramen ovale and epidural peg electrodes, none had scalp VEM; ^b^all 5 patients have had scalp VEM as their first VEM; ^c^all 6 had subdural or depth electrodes

During the study period, 116 patients underwent a total of 165 VEM with a maximum of three separate VEM per individual patient, and 20 patients were eventually found to be ineligible for resection. Reasons for non-candidacy for resection are given in Supplemental Material Fig. 1. When analysing the remaining 96 patients’ last decision during the study period, 51 patients declined and 45 agreed on the given recommendations for either resection or further intracranial or scalp VEM (53% vs 47%). Splitting this up, 40 patients were offered resective surgery of whom 5 declined (12.5%) all of which after scalp VEM. Among the 74 patients offered intracranial EEG, 29 declined (39.2%); further scalp VEM was recommended to 31 patients of whom 17 declined (54.8%) (see Fig. 1).

Patients were individually and openly asked for reasons why they declined the given recommendation (*n = *51); multiple answers were possible. The most frequent reason was a general fear of brain surgery (*n = *30, 59%), followed by currently perceived low seizure frequency (*n = *11, 22%), fear of peri- or postoperative complications (*n = *9, 18%), and assumed low probability of seizure freedom by patients (*n = *9, 18%).

### Predictors to decline recommendations

Living status and social support are factors that may influence decision-making. Among those that declined further VEM or surgery (*n = *51) compared to those that agreed (*n = *45), though on a descriptive level, more patients were living alone (17, 33% vs. *n = *9, 20%; *p = *0.245) and less had children living in the same household (*n = *8, 16% vs. *n = *10, 22%; *p = *0.867), and neither of these comparisons was statistically significant using univariate analysis (see Supplemental Material Table 1).

In a multivariate analysis, patients’ decline of recommended resective surgery, further scalp, or intracranial VEM diagnostics was independently predicted by less epilepsy-related fear (OR 0.43; 95% CI 0.210–0.873, *p = *0.02) as assessed by the PESOS questionnaire, and there was a trend towards younger age (OR 0.96; 95% CI 0.915–1.000; *p = *0.051) (Table 1).

## Discussion

Underutilization of epilepsy surgery is highly complex and comprises the referral as well as the treatment gap both of which are still insufficiently understood. Important factors include knowledge deficits and misconceptions on both physicians’ and patients’ sides [12].

In this work, we aimed to investigate reasons for patients’ decline on given recommendation for either resective surgery or further presurgical VEM diagnostics with scalp or intracranial electrodes. Overall, rates of decline and agreement by patients in our study are in line with previous studies [4–6]: Twenty-eight percent declined the recommendation to undergo resective surgery after scalp VEM, and 39% declined to undergo intracranial EEG (Fig. 1). It is noticeable that none of the patients declined resective surgery after intracranial EEG. The most likely reason is that in our centre, almost all patients with intracranial EEG receive subdural electrodes, which means that electrode explantation and resective surgery are done during the same operation. As our patients are prepared in advance of that, decline on recommendation for resection after intracranial EEG is very rarely seen. This is likely different after recording with depth electrodes when their removal and a possible resection are performed at different time points. The highest rate of decline was for the recommendation of repeat scalp VEM (55%). This may be explained by a combination of frustration about unclear results and uncertainty about the general possibility of epilepsy surgery. The most frequent reasons for repeating scalp VEM are a lack of seizures despite reduction of antiseizure medication or diffuse seizure onset, and thus, patients might not have confidence that a repeat VEM would yield more definite results. Furthermore, a previous retrospective study from our centre has demonstrated that decline of further presurgical VEMs with scalp electrodes was independently associated with a lower life-time number of antiseizure medications [6]. Thus, patients may hope that further antiseizure medication results in seizure freedom.

The most frequent reason for decline by patients, regardless of the given recommendation, was general fear of brain surgery. This mirrors results from previous work, where patients’ perceptions and knowledge about and decisions towards epilepsy surgery were investigated [9, 13–15].

Among the psychosocial variables that we included into our multivariate analysis, less epilepsy-related fear predicted patients’ decline. This variable is an item from the standardised PESOS questionnaire and includes 11 subitems asking patients regarding fear about their seizures [11]. It is understandable that patients with fewer negative experiences in daily life due to their epilepsy and resulting lower epilepsy-related fear may weigh fears towards surgery more strongly in their decision-making. A small prospective study about patients’ decision-making in presurgical assessment using a self-designed questionnaire showed similar findings compared to our results: Higher fear of surgery in general and less fear of embarrassment from seizures in public were significantly linked to patients’ decline to undergo epilepsy surgery [9].

Another psychosocial factor possibly influencing patients’ decisions is employment status which turned out to be not a significant independent variable in the multivariate analysis (Table 1). This contradicts previous work that showed that being employed led to a higher rate of patients declining epilepsy surgery [7]. Another important factor may be patients’ social support system. As part of the PESOS questionnaire, we had assessed patients’ living/social status (see Supplementary Material Table 1). Among those that declined further VEM or surgery, more were living alone (33% vs. 20%) and less had children living in the same household (16% vs. 22%), though differences were not statistically significant. One could argue that caring responsibilities could also be a factor that leads patients to decline recommendations to proceed to surgery or further diagnostic VEM due to being absent from home. However, in the end, this is a fairly complex variable to assess. More insight is needed to address fears, perceptions and influencing psychosocial variables better when counselling patients on epilepsy surgery.

Compared to previous work, one of the strengths of our study is its prospective design that assesses both biological and psychosocial variables. Previous retrospective studies relied mostly on either of these aspects. Furthermore, we enquired about patients’ personal views as to why they declined physicians’ recommendations.

A limitation of our study is its monocentric design and, thus, the resulting relatively small number of patients which may hamper generalisability of the findings.

In summary: Patients declining epilepsy surgery despite eligibility need more targeted counselling on the risks and benefits which also addresses their individual fears. Beyond high rates of seizure freedom, quality of life increases, and morbidity and mortality decrease postoperatively significantly compared to continued pharmacotherapy without surgery [2, 15, 16]. The current findings give valuable insight into patients’ decision-making during presurgical assessment. Further research in larger and possibly multicentre cohorts is needed to understand influencing psychosocial factors better.

## Supplementary Information

Below is the link to the electronic supplementary material.

Supplementary file1 (DOCX 16 kb)

Supplementary file2 (DOCX 32 kb)

Supplementary file3 (DOCX 16 kb)

## Data Availability

The data that support the findings of this study are available from the corresponding author upon reasonable request due to privacy issues of clinical data. A formal data sharing agreement is needed. All data relevant to the study are included in the article or uploaded as online supplemental information.
