# Supplementary material for: Predictors and reasons for epilepsy patients to decline surgery: a prospective study
Source: J Neurol. 2022 Dec 6;270(4):2302–7. doi: 10.1007/s00415-022-11510-3 (PMC10025225; doi:10.1007/s00415-022-11510-3)
Supplement: Supplementary file 1 — Supplementary file1 (DOCX 16 kb) [file 415_2022_11510_MOESM1_ESM.docx]

**Supplemental material - Figure 1**.

Reasons for decision of interdisciplinary epilepsy surgery meeting that surgery was non-feasible (n=20 patients); multiple reasons per patient were possible; other included diverse individual reasons (e.g. care taking responsibilities, fear of losing job).
