# Supplementary material for: Predictors and reasons for epilepsy patients to decline surgery: a prospective study
Source: J Neurol. 2022 Dec 6;270(4):2302–7. doi: 10.1007/s00415-022-11510-3 (PMC10025225; doi:10.1007/s00415-022-11510-3)
Supplement: Supplementary file 2 — Supplementary file2 (DOCX 32 kb) [file 415_2022_11510_MOESM2_ESM.docx]

**Supplemental material - Table 1 – Clinical variables**

|  | **All** | **Decline^a^** | **Agreement^a^** |
| --- | --- | --- | --- |
| **N** | 116 | 51 | 45 |
| **Sex** (female, n (%)) | 55 (47) | 26 (51) | 23 (51) |
| **Age at last scalp VEM**, years (median, IQR) | 32 (27 - 41) | 33 (29 - 44) | 31 (24 - 38) |
| **Age at epilepsy onset**, years (median, IQR) | 13 (18 - 21) | 13 (8 - 25) | 14 (8 - 21) |
| **Duration of epilepsy**, years (median, IQR) | 15 (8-23) | 18 (10 - 24) | 16 (8 - 22) |
| **Number of current ASM,** n (median, IQR) | 2 (2 - 3) | 2 (1 - 3) | 2 (2 - 3) |
| **Number of lifetime ASM,** n (median, IQR) | 5 (3 - 7) | 5 (3 - 7) | 5 (3 - 7) |
| **Seizure type** (n (%)) |  |  |  |
| focal aware seizures | 84 (73) | 36 (71) | 38 (84) |
| focal impaired awareness seizures | 105 (90) | 44 (86) | 40 (89) |
| focal to bilateral tonic-clonic-seizures | 93 (81) | 43 (84) | 35 (78) |
| psychogenic non-epileptic seizures | 14 (12) | 3 (6) | 6 (13) |
| **Lateralisation of ictal EEG** (n (%)) |  |  |  |
| right | 30 (26) | 9 (18) | 21 (47) |
| left | 30 (26) | 11 (21) | 21 (47) |
| bilateral | 21 (28) | 7 (14) | - |
| diffuse | 22 (19) | 17 (33) | 2 (4) |
| NA | 13 (11) | 7 (14) | 1 (2) |
| **Localisation of ictal EEG** (n (%)) |  |  |  |
| temporal | 60 (52) | 22 (43) | 33 (73) |
| frontal | 21 (18) | 11 (21) | 5 (11) |
| parietal/occipital | 3 (3) | - | 3 (7) |
| multifocal | 2 (2) | 2 (4) |  |
| multilobar | 7 (6) | 1 (2) | 2 (4) |
| uncertain | 10 (9) | 8 (16) | 1 (2) |
| NA | 13 (11) | 7 (14) | 1 (2) |
| **MRI lesion** (n (%)) |  |  |  |
| no | 42 (36) | 18 (35) | 16 (36) |
| yes | 48 (42) | 19 (37) | 24 (53) |
| equivocal | 26 (22) | 14 (28) | 5 (11) |
| **Lateralisation of MRI lesion** (n (%)) |  |  |  |
| right | 23 (20) | 8 (24) | 8 (27) |
| left | 36 (31) | 18 (55) | 15 (52) |
| bilateral | 15 (13) | 7 (21) | 5 (17) |
| **Localisation of MRI lesion** (n (%)) |  |  |  |
| temporal | 49 (43) | 23 (70) | 20 (69) |
| frontal | 4 (4) | 2 (6) | 2 (7) |
| parietal/occipital | 10 (9) | 10 (15) | 5 (17) |
| multifocal | 11 (10) | 3 (9) | 2 (7) |
| **MRI pathology** (n (%)) |  |  |  |
| mesial temporal sclerosis | 10 (13) | 3 (9) | 6 (21) |
| focal cortical dysplasia | 9 (12) | 3 (9) | 6 (21) |
| gliosis/focal atrophy | 11 (15) | 4 (12) | 5 (17) |
| lowgrade tumor | 3 (4) | 2 (6) | 1 (3) |
| arteriovenous malformation | 2 (2) | 2 (6) | - |
| polymicrogyria | 2 (2) |  | 2 (7) |
| heterotopia | 6 (5) | 3 (9) | 1 (3) |
| others | 26 (49) | 14 (42) | 5 (17) |
| dual | 5 (7) | 2 (6) | 3 (10) |
|  |  |  |  |
| **Epilepsy syndrome** (n (%)) |  |  |  |
| temporal lobe epilepsy | 58 (50) | 22 (43) | 33 (73) |
| extra-temporal lobe epilepsy | 22 (19) | 9 (18) | 8 (18) |
| uncertain region of seizure origin | 36 (31) | 20 (39) | 4 (9) |
| **Intracranial EEG** (n (%)) | 37 (32) | 3 (6) | 23 (51) |
| **Resective surgery** (n (%)) | 35 (30) | 0 | 35 (77) |
| after scalp VEM | 13 (37) | 0 | 13 (37) |
| after intracranial VEM | 22 (63) | 0 | 22 (63) |
| **Outcome one year post surgery** (ILAE) |  |  |  |
| ILAE 1 | 21 (60) | - | 21 (60) |
| ILAE 2 | 1 (3) | - | 1 (3) |
| ILAE 3 | 3 (9) | - | 3 (9) |
| ILAE 4 | 6 (17) | - | 6 (17) |
| ILAE 5 | 4 (11) | - | 4 (11) |
| ILAE 6 | - | - | - |
| **Histopathology** |  |  |  |
| mesial temporal sclerosis | 12 (34) | - | 12 (34) |
| gliosis | 10 (29) | - | 10 (29) |
| focal cortical dysplasia | 4 (11) | - | 4 (11) |
| cortical malformation of development | 1 (3) | - | 1 (3) |
| lowgrade tumor | 3 (9) | - | 3 (9) |
| other | 2 (6) | - | 2 (6) |
| not available | 3 (9) | - | 3 (9) |
| **Outcome one year after last VEM in non-resected patients** (ILAE) |  |  |  |
| ILAE 1 | 6 (7) | 1 (2) | 3 (30) |
| ILAE 2 | 3 (4) | 3 (6) | - |
| ILAE 3 | 4 (5) | 4 (8) | - |
| ILAE 4 | 17 (21) | 11 (22) | - |
| ILAE 5 | 49 (61) | 30 (59) | 7 (70) |
| ILAE 6 | - | - | - |
| NA | 2 (2) | 2 (4) |  |
| **Psychiatric comorbidities** (n (%)) |  |  |  |
| none | 77 (66) | 37 (72) | 29 (64) |
| depression/anxiety | 13 (11) | 7 (14) | 5 (11) |
| personality disorder | 3 (3) |  | 2 (4) |
| autism | 2 (2) | 1 (2) |  |
| psychosis | 2 (2) |  | 3 (7) |
| psychogenic non-epileptic seizures | 8 (7) | 1 (2) | 1 (2) |
| substance abuse | 2 (2) | 1 (2) | 2 (4) |
| multiple | 6 (5) | 3 (6) | 3 (7) |
| other | 3 (3) | 1 (2) |  |
| **Patients’ profession** (n (%))^b^ |  |  |  |
| employee | 38 (32) | 22 (43) | 14 (31) |
| student | 13 (11) | 4 (8) | 5 (11) |
| trainee | 6 (5) | 1 (2) | 4 (9) |
| seeking work | 27 (24) | 10 (20) | 14 (31) |
| sheltered workshop | 7 (6) | 2 (4) | 2 (4) |
| reduced earning capacity pension | 21 (18) | 11 (22) | 4 (9) |
| dependent on partner’s/ families’ income | 4 (3) | 1 (2) | 2 (4) |
|  |  |  |  |
|  |  |  |  |
|  |  |  |  |
| **Patients’ social status** (n (%)) |  |  |  |
| Living alone | 31 (27) | 17 (33) | 9 (20) |
| Single parent | 2 (2) | 2 (4) | 1 (2) |
| Living with partner | 17 (15) | 8 (16) | 7 (16) |
| Living with partner and children | 15 (13) | 6 (12) | 9 (20) |
| Living with parent/ family member | 22 (19) | 8 (16) | 10 (22) |
| Assisted living | 1 (1) |  | 1 (2) |
| Other | 6 (5) | 2 (4) | 3 (7) |
| NA | 22 (19) | 8 (16) | 5 (11) |

n: number of patients; VEM, video-EEG monitoring; IQR: interquartile range; ASM: antiseizure medication; NA: not available; ILAE: International League Against Epilepsy.
^a^ Patients’ last decision during study period either for or against given recommendation to either resective surgery, intracranial EEG or further scalp VEM; excluding n= 20 patients where surgery was deemed non-feasible by the interdisciplinary epilepsy surgery meeting. ^b^For the multivariate analysis, we created the dichotomized variable employment status and subsumed patients’ professions into two categories: employed includes employees, students, trainees and public servants and unemployed includes job-seekers and people without own income (dependent on partner’s/ families’ income, working in sheltered workshop, reduced earning capacity pension).
