# Supplementary material for: Predictors and reasons for epilepsy patients to decline surgery: a prospective study
Source: J Neurol. 2022 Dec 6;270(4):2302–7. doi: 10.1007/s00415-022-11510-3 (PMC10025225; doi:10.1007/s00415-022-11510-3)
Supplement: Supplementary file 3 — Supplementary file3 (DOCX 16 kb) [file 415_2022_11510_MOESM3_ESM.docx]

**Supplemental material - Table 2 – Results PESOS questionnaire**

|  | **Patients’ Decision** |  |
| --- | --- | --- |
|  | **Decline** | **Agreement** |
| **Restrictions in daily life**, n (%) | 43 | 40 |
| very strong (≥76%) | 1 (2) | 1 (3) |
| strong (51-75%) | 8 (19) | 9 (22) |
| somewhat (26-50%) | 13 (30) | 19 (48) |
| very little (≤25%) | 19 (44) | 11 (27) |
| none | 2 (5) | 0 |
| **Epilepsy-related fear**, n (%) | 43 | 40 |
| strong (=100%) | 2 (5) | 5 (12) |
| somewhat (66-99%) | 14 (33) | 19 (48) |
| very little (33-65%) | 24 (56) | 15 (37) |
| none (≤ 32%) | 3 (7) | 1 (3) |
| **Overall satisfaction with therapy, n (%)** | 43 | 41 |
| very satisfied | 2 (5) | 8 (20) |
| satisfied | 24 (56) | 15 (37) |
| unsatisfied | 16 (37) | 17 (41) |
| very unsatisfied | 1 (2) | 1 (2) |

PESOS: Performance, socio-demographic aspects, subjective evaluation; n: number of patients.
The subscale *restrictions in daily life* includes questions regarding mobility and self-reliance; relationships, family and friends and physical and emotional well-being; *Epilepsy related-fear* includes questions regarding fear to have a seizure, fear regarding negative effects of seizures on relationships or employment, fear of being injured during a seizure.

Overall scores were ranked within the group and then categorised as has been described in the original publication (*Pfaefflin TWMaM. Evaluating comprehensive care: description of the PESOS and its psychometric properties In: M. Pfaefflin RTF, R. Thorbecke, U. Specht, P. Wolf ed. Comprehensive Care For People With Epilepsy. Eastleigh, England: John Libbey & Company LTD 2001:pp. 319-40*).
